# Profiles of Family Stressors Among Low-Income Families with Young Children

**DOI:** 10.1007/s10995-025-04061-2

**Published:** 2025-02-25

**Authors:** Laura M. Justice, Britt Singletary, Hui Jiang, Kammi K. Schmeer

**Affiliations:** 1 Crane Center for Early Childhood Research & Policy, The Ohio State University, 175 E. 7th Avenue, Columbus, OH 43201 USA; 2 Department of Sociology, The Ohio State University, Columbus, OH USA

**Keywords:** Disrupted parenting, Economic hardship, Economic pressure, Family stress model, Psychological distress, Relationship problems

## Abstract

**Objectives:**

This study examined profiles of family stressors, based on the five dimensions of the Family Stress Model (economic hardship, economic pressure, parental psychological distress, interparental relationship problems, and disrupted parenting) among low-income families with young children. We aimed to validate the model with a sample of low-income families and then to determine whether there were reliable profiles of families on the five dimensions.

**Methods:**

Mothers completed questionnaires when children were between six and 15 months old to capture background information and 14 key indicators of the five Family Stress Model dimensions. Our analytical sample comprised 353 families that provided data for at least one key indicator. We conducted confirmatory factor analysis (CFA) to statistically validate the Family Stress Model, then explored distinct profiles using latent profile analyses (LPA), and examined how profile membership correlated with family characteristics.

**Results:**

CFA model fit indices indicated a good fit of the data relative to the theoretical model. LPA revealed three distinct profiles of stressor among families, consistent with low, medium, and high patterns of stressors. Families in the high-stress profile experienced larger household numbers, higher maternal loneliness, reduced social connectedness, and higher reports of unplanned pregnancy.

**Conclusions for practice:**

Findings show applicability of the Family Stress Model to low-income families with young children, and indicate that these families are diverse in terms of the stressors they experience, characterized by three distinct profiles. In this regard, low-income families should not be viewed monolithically, but rather as experiencing variability in the stressors they face.

**Supplementary Information:**

The online version contains supplementary material available at 10.1007/s10995-025-04061-2.

## Significance

What is already known on this subject?

What this study adds?

Economic hardship introduces a variety of stressors into one’s home, which may disrupt young children’s development. The Family Stress Model specifies the impact of economic hardship on introducing stressors into households via increases in economic pressure, parental psychological distress, interparental relationship problems, and disrupted parenting.

This study examines the validity of the Family Stress Model for low-income families with young children, and examines whether there are profiles of stressors that characterize subgroups of families. We show that 55% of low-income families with young children show profiles of medium- to high-stressors and we hypothesize that in these profiles of homes, children are most vulnerable to adverse developmental outcomes.

**Supplementary Information:**

The online version contains supplementary material available at 10.1007/s10995-025-04061-2.

Approximately one in seven children in the United States resides in a household with an annual income below the nation’s poverty threshold (Creamer et al., 2022). Children in families experiencing economic hardship are vulnerable for a wide range of adversities that transcend early childhood to adolescence. These include, for instance, vulnerability for developmental delays and disabilities (Norbury et al., 2016) and elevated rates of social, behavioral, and mental-health challenges (Bernard et al., 2015); childhood poverty is also associated with adversities well into adulthood, such as lower post-secondary education participation and employment (Duncan & Magnuson, 2013). In part, the consistently observed interrelations between household poverty and children’s outcomes are reflective of the ways in which economic hardship disrupts the family’s functioning, as theorized in the Family Stress Model (Conger & Conger, 2002).

The Family Stress Model is a well-validated theoretical model that specifies the way in which economic hardship contributes to adverse outcomes for children and youth (Conger & Conger, 2002; Conger et al., 2002). This model proposes that the impacts of poverty, or economic hardship, on children and youth is through its association with distinct dimensions of stress. More specifically, *economic hardship*, such as an inability to pay bills or rent, creates *economic pressure*, or feelings of psychological or emotional burden caused by economic hardship. These pressures lead to vulnerabilities within the family structures and processes, contributing to *psychological distress* (e.g., anxiety, depression), *inter-personal relationship problems*, and *disrupted parenting*. Collectively, the Family Stress Model encompasses five dimensions of stressors in an overarching model of how poverty disrupts the household.

In the present study, our first aim was to determine the applicability of the Family Stress Model to documenting multi-dimensional stressors within the homes of low-income families with very young children. The Family Stress Model identifies five distinct dimensions of stressors (i.e., economic hardship, economic pressure, psychological distress, inter-personal relationship problems, and disrupted parenting) as the pathways through which poverty influences the development of young children, but the validity of this model with low-income families with young children has not occurred. The second aim is to explore whether there are distinct profiles of low-income families with respect to the model’s dimensions. We hypothesized that low-income families are not monolithic in the stressors they encounter, and that there are different profiles that reliably characterize low-income families. To address this second aim, we employed a person-centered approach, latent profile analysis, to account for the potentially heterogeneous nature of our sample with respect to the five dimensions of stressors. We also explored correlates of the profiles with respect to family demographic factors, including maternal education and employment and child gender, among others; by exploring these correlates, the study may serve to identify family characteristics that are salient to any emergent profiles.

## Methods

### Study Participants

All study procedures adhered to prevailing ethical principles for human-subjects research, and were approved by an Institutional Review Board (IRB Protocol: 2019B0220 - SMALL Talk: Study of Milestones to Advance Language Learning). A total of 356 mothers with children aged 6–15 months were enrolled and provided informed consent to participate in a longitudinal study across two enrollment periods – the original enrollment period from October 2019 through December 2020 (*n* = 337; when focal children were 6–12 months old) and a replenishment enrollment period from August 2021 through November 2021 (*n* = 19; when focal children were 13–15 months old, corresponding to the second timepoint of data collection for the rest of the sample [*n* = 337]) to account for attrition of the originally enrolled sample during their first year in the study). Pre-pandemic recruitment occurred in-person at local centers and events serving low-income mothers (*n* = 20 mother-infant dyads); after the onset of the pandemic, recruitment occurred remotely via responses to text advertisements sent through local Women, Infant, and Children (WIC) centers to WIC-eligible families (*n* = 336 mother-infant dyads).

Interested mothers completed an eligibility screener prior to consenting to join the study; eligibility requirements included that mothers were aged 18 years or older, were comfortable speaking and reading English, did not have plans to move out of the area in near future, and their focal children were 6–15 months old, from singleton births, born at 35 weeks or greater gestation, and had not been diagnosed with any significant disability at or around birth. Additionally, to ensure our sample represented a low-income population, eligible families were currently receiving governmental assistance (e.g., WIC, food stamps, CareSource, Medicaid, or housing subsidies) or resided in households less than 200% of the 2019 federal poverty level for their household size. Table 1 provides details of the sample.

**Table 1:** Descriptive information for the analytical sample (*n* = 353)

	Valid *N*	% or Mean	SD	Min	Max
Characteristics of mothers, children, and families					
Child age in months at enrollment	353	8.43	2.03	4	16
Child gender: Female	353	50%			
Child race	339				
White		25%			
Black/African American		50%			
Asian		2%			
Other		6%			
Multiracial		17%			
Child ethnicity: Hispanic	341	11%			
Mother age in years (year 1)	350	29.34	5.68	18	44
Mother race	346				
White		34%			
Black/African American		50%			
Asian		3%			
Other		6%			
Multiracial		7%			
Mother ethnicity: Hispanic	349	7%			
Mother’s highest level of education	349				
Less than high school		13%			
High school diploma or GED		30%			
Some college but no degree		29%			
Associate’s degree		7%			
Bachelor’s degree		15%			
Graduate or professional degree		5%			
Annual household income (in $1000)	341	25.81	17.59	5	95
Number of people in household	353	4.53	1.57	2	11
Primary home language: English	353	88%			
*Indicators of the Family Stress Model*					
Income-to-needs ratio	341	0.95	0.65	0.09	3.54
Access to health insurance	353				
No health insurance		6.2%			
Medicaid/Government-aided insurance		75.9%			
Private or multiple insurance(s)		17.8%			
Material resources	337	0.71	0.29	0.00	1.00
Food insecurity	338	0.10	0.16	0.00	0.86
Household chaos	339	2.43	0.59	1.17	4.08
Maternal depression	308	0.52	0.64	0.00	3.33
Maternal anxiety	334	0.57	0.64	0.00	3.00
Maternal stress	273	1.43	0.75	0.00	3.90
Maternal experience with discrimination	228	0.80	0.87	0.00	4.33
Frequent family conflict	331	71.6%			
Relationship conflict	236	2.18	0.56	1.25	4.38
Parenting self-efficacy	342	9.09	0.91	4.84	10.00
Mother-child attachment	325	4.77	0.29	3.19	5.00
Parenting aggravation^1^	333	3.37	0.44	1.78	4.00

^1^ Unlike the other indicators included wherein higher scores represent more stress/problems, higher score represents lower parenting aggravation

### Study Procedure

The present study used data collected at timepoint 1 (i.e., the time of enrollment for the originally enrolled sample, *n* = 337; January 2020 to March 2021) and timepoint 2 (representing the time of enrollment for the replenishment sample, *n* = 19), when children were aged 6–12 months and 13–15 months, respectively. Timepoint 1 data were initially captured in-person using a combination of interview-based and self-reported questionnaires with a trained assessor and an iPad hosting the Qualtrics secure software application (*n* = 20). Following the onset of the pandemic, timepoint 1 data collection transitioned to a phone call with a trained assessor to obtain interview-based questionnaires followed by the mother completing personalized links sent by email using Qualtrics for more sensitive self-reported questionnaires (*n* = 317). Not all these mothers also completed timepoint 1 emailed surveys (*n* = 13 did not finish), resulting in variation in sample sizes for measures administered this way at this timepoint. Additionally, as the replenishment sample (*n* = 19 mother-child dyads) enrolled after timepoint 1, and they did not complete any measures for timepoint 1.

We attempted to capture timepoint 2 data from all originally enrolled (*n* = 337) and replenishment (*n* = 19) mother-child dyads using phone call and email methods (August 2020 to November 2021), but only 305 mothers agreed to complete the phone call portion of this timepoint. Additionally, not all these mothers also completed timepoint 2 emailed surveys (*n* = 19 did not finish), resulting in variation in sample sizes for measures at this timepoint as well. Mothers were sent $35 in gift cards at each timepoint following completion of both the phone and emailed surveys.

### Measures

Timepoint 1 and timepoint 2 both included surveys administered by trained assessors (either in-person or by phone), in addition to surveys administered via email using personalized links. Assessor-led surveys took 30–60 min to complete, whereas emailed surveys took another 30–60 min to complete, but could be completed at any time convenient to the participant in the weeks following the phone call.

Taken together, these surveys captured a range of content areas, such as child development, behavior, and temperament; maternal background, attachment, and health; and maternal perceptions of parenting. Here, we describe only the primary measures used in the current study, namely family characteristics and measures representing the five dimensions of the Family Stress Model. Note that a thorough description of each measure is presented in Supplementary Material, whereas here we summarize these measures.

Notably, for the originally enrolled sample (*n* = 337), when data are referenced as having been collected at enrollment, this occurred at timepoint 1 of the overall study (when children were 6–12 months old). Alternatively, for the replenishment sample (*n* = 19), when data are referenced as having been collected at enrollment, this occurred at timepoint 2 of the overall study (when children were 13–15 months old), as this was the current timepoint at which the replenishment dyads joined the study.

### Family Characteristics

Mothers reported on a variety of child and family characteristics at enrollment, including child sex and age; total number of people in household; primary home language; mother’s age, ethnicity, race, educational attainment, and employment. Additionally, at enrollment, we assessed mother’s relationship status; whether the mother’s pregnancy with the focal child was planned; and whether the focal child’s father provided regular support. At enrollment, mother’s social connectedness with friends and with community were measured using questions from the Berkman-Syme Social Network Index (Berkman, 1977). At timepoint 2, mother’s loneliness was measured using the UCLA Loneliness Scale version 3 (Russell, 1996), which assesses subjective feelings of social isolation and loneliness.

### Dimensions of the Family Stress Model

The five dimensions of the Family Stress Model were assessed using 14 distinct indicator measures to capture economic hardship, economic pressure, maternal psychological distress, relationship problems, and disrupted parenting.

*Economic hardship*, or the financial constraints a family experiences, was characterized by three measures collected at enrollment: income-to-needs ratio, access to health insurance, and availability of financial resources. For the latter, financial resources included access to a (1) checking account, (2) savings account, (3) credit card, and (4) driver’s license (as an indicator of having access to obtain household needs).

*Economic pressure*, or the psychological and emotional burden associated with economic hardship, was characterized by two measures at enrollment: food insecurity and household chaos. Food insecurity was measured using the USDA Household Food Security Scale (Bickel et al., 2000), comprising 18 items that assess a family’s ability to meet the food needs of their family. Household chaos was measured using a shortened version of the Confusion, Hubbub, and Order Scale (CHAOS; Matheny et al., 1995), comprising six items that assess the overall stability of the home atmosphere.

*Maternal psychological distress*, reflecting the emotional well-being of the mothers, was characterized by four measures: maternal depression, anxiety, stress, and experience with discrimination, with maternal depression assessed only at timepoint 1, maternal anxiety at both timepoint 1 and 2 (scores averaged across timepoints in the event that dyads had data for both timepoints), and maternal stress and experience with discrimination at timepoint 2. Maternal depression was measured using the Center for Epidemiological Studies of Depression Scale – Revised Form (CESD-R; Eaton et al., 2004), which comprises 20 items that assess symptoms of depression. Maternal anxiety was measured using the Generalized Anxiety Disorder 7-Item Scale (GAD-7; Löwe et al., 2008; Spitzer et al., 2006), which consisted of seven items that assess symptoms of anxiety. Maternal stress was measured using the Perceived Stress Scale (PSS; Cohen, 1994; Cohen et al., 1983), consisting of 10 items that assess the participant’s perception of stress experienced in their day-to-day life. Maternal experience with discrimination was measured using the Everyday Discrimination Scale (EDS; Williams et al., 1997), comprising nine items that assess how often the participant experiences different topics of discrimination.

*Relationship problems* capture the degree of conflict within the household environment and were assessed with two measures at enrollment: family conflict and relationship conflict. Family conflict was measured using the Family Environment Scale – Conflict Subscale (FES; Moos & Moos, 2009), comprising nine items that assess family conflict and negative communication. Relationship conflict was measured using a subset of questions from the Braiker-Kelley Relational Intimacy Scale – Conflict-Negativity Subscale (Braiker & Kelley, 1979), namely four items that assess behavioral conflict and negative communication within their relationship. If participants reported that they were not in a relationship, these four items were skipped and no scale score was generated.

*Disrupted parenting* represents the overall quality of the parent-child relationship and interactions, and was characterized by three measures at enrollment: parenting self-efficacy, mother-child attachment, and parenting aggravation. Parenting self-efficacy was measured using the Parental Cognitions and Conduct Toward the Infant Scale – Parental Self-Efficacy Subscale (PACOTIS; Boivin et al., 2005), consisting of six items that assessed the perception of the mother’s feelings of effectiveness as a parent. Mother-child attachment was measured using the Maternal Postnatal Attachment Questionnaire – Quality of Attachment Subscale (MPAQ; Condon & Corkindale, 1998), consisting of nine items that assess the quality of attachment between a mother and her child. Parenting aggravation was measured using the Fragile Families Aggravation in Parenting Questions (The Fragile Families and Child Wellbeing Survey, 2005), consisting of nine items that assess how parenting makes the participant feel.

### Analytical Overview

Our analytical sample was comprised of 353 families that provided data for a minimum of one out of the 14 family stress measures (see Table 1). As a preliminary analysis, we examined the descriptive statistics of the 14 family stress measures and calculated pairwise correlations between them. Pearson correlation coefficients were used to measure the strength of correlation between continuous or binary indicators, while Spearman correlation coefficients were employed in instances where at least one indicator was ordinal. We then conducted confirmatory factor analysis to statistically validate the Family Stress Model, encompassing five distinct factors: economic hardship, economic pressure, maternal psychological distress, relationship problems, and disrupted parenting, via 14 key indicators. Guided by modification indices and theoretical considerations, we introduced a covariance term between maternal depression and maternal anxiety. To determine how well the factor models fit the data, we examined the following fit indices: Root Mean Square Error of Approximation (RMSEA; < 0.05 good fit, < 0.08 acceptable fit), Comparative Fit Index (CFI; > 0.95 good fit, > 0.90 acceptable fit), and Tucker-Lewis Index (TLI; > 0.95 good fit, > 0.90 acceptable fit). Once the factor model was validated, we then derived factor scores representing composites for each of the five dimensions of family stressors.

Subsequently, to explore whether there were distinct profiles of low-income families as a function of family stressors, we employed Latent Profile Analysis (LPA) using the factor scores extracted from the factor model. LPA is a statistical method to identify subgroups (i.e., profiles) of similar cases based on multiple attributes measured on continuous scales (Clogg, 1995). Given a certain number of subgroups or profiles, each case is evaluated to see how likely it is to belong to each subgroup. In our analyses, we tested models with different numbers of profiles (two, three, four, and five) to examine which one best represented the data. To decide, we inspected various fit statistics such as the Akaike Information Criterion (AIC; lower is better), Bayesian Information Criterion (BIC; lower is better), Sample Size Adjusted Bayesian Information Criterion (SSABIC; lower is better), and best log-likelihood. The log likelihood calculates how likely the data are observed as they are given the model, and a higher log likelihood value indicates that the model fits the data better. We also conducted Lo-Mendell-Rubin (LMR) Adjusted Likelihood Ratio Test to examine whether adding one more profile can significantly change model fit (Lo et al., 2001; Vuong, 1989). Additionally, we examined entropy to see how distinct the profiles are different from one another (Celeux & Soromenho, 1996). Entropy values can range from 0 to 1, and higher entropy means that the groups are more distinct from each other. As a rule of thumb, an entropy value of 0.8 or above is considered good, indicating clear separation between profiles. Finally, we examined the summarized attributes of each subgroup to see if they make theoretical sense. We found that the model with three profiles was the best fit, statistically and theoretically for describing patterns of family stressors for our sample.

We then explored the association between family profile membership and a variety of family characteristic covariates. In addition to calculating the descriptive statistics of each family covariate across profiles, we conducted ANOVA to examine differences between profiles for continuous variables, and Chi-squared tests to assess cross-profile differences in categorical variables.

### Missing Data

Missing data ranged from 0 to 23% for the majority of the 14 family stress measures within the analytical sample, with the exception of relationship conflict (33%) and maternal experience with discrimination (35%). As noted above, participants who reported that they were not in a relationship skipped the items of relationship conflict, leading to a higher percentage of missing data. The items of maternal experience with discrimination had lower response rates compared to other scales.

To utilize all available data, we employed full information maximum likelihood (FIML) technique in CFA and LPA to treat missing data across all variables (Arbuckle et al., 1996), regardless of the format of survey methods or the reasons of missingness. Instead of filling in missing values or discarding cases with missing data, FIML uses all available data to estimate model parameters directly. For example, for the dimension of Relationship Problems, if a participant had missing data on relationship conflict, her reported data on family conflict would be used to extract information on that dimension. Whereas FIML assumes that data are missing completely at random (MCAR; missingness is due to random chance and unrelated to any variables) or missing at random (MAR; missingness is related to observed variables included in the model), it is usually reasonable to assume MAR under most circumstances, and FIML is robust to mild deviation from MAR (Collins et al., 2001; Schafer & Graham, 2002).

## Results

### Indicators of the Five Dimensions of the Family Stress Model

Table 1 presents the descriptive statistics of the 14 selected indicators of the five dimensions of the Family Stress Model: economic hardship, economic pressure, maternal psychological distress, relationship problems, and disrupted parenting. Notably, the sample consists of families with a mean income-to-needs ratio (INR) of 0.95, indicating that average family income falls below the poverty threshold. Moreover, 26% of the families reported INRs lower than 0.50, 60% had INRs lower than 1, whereas 92% had INRs lower than 2. Only 1% of the sampled families reported INRs at or exceeding 3.

These families also reported higher levels of economic pressure, including elevated food insecurity and household chaos. Additionally maternal stress, an indicator of maternal psychological distress, was elevated for these families. Other key indicators, such as depression, anxiety, conflict, and parenting attitudes were less elevated.

Indicators of family economic hardship showed low correlation with other indicators (mean correlation = − 0.01), suggesting that economic constraints may be somewhat distinct from other stressors (see Table 1 in Supplementary Materials). On the other hand, indicators of all other dimensions displayed higher cross-dimensional correlations. For example, indicators related to maternal psychological stress exhibited a small to moderate correlation with those representing economic pressure (mean correlation = 0.22).

### Validation of the Five-Dimensional Model of Family Stress

To validate the five-dimensional Family Stress Model using our 14 key indicators, we conducted confirmatory factor analysis; results are presented in Table 2. Factor loadings ranged from 0.38 to 0.84, and model fit indices indicated a good fit of the data relative to the theoretical model: RMSEA = 0.037, with a 95% confidence interval of 0.020 to 0.052; CFI = 0.959; and TLI = 0.943. The Chi-square value was 97.65 (degree of freedom = 66, *p* =.007).

**Table 2:** Confirmatory factor analyses of five-dimensional the family stress model

	Economic hardship^4^		Economic pressure		Maternal psychological distress		Relationship problems		Disrupted parenting^5^		*R* ^2^
	Loading	S.E.	Loading	S.E.	Loading	S.E.	Loading	S.E.	Loading	S.E.	
Income-to-needs ratio	0.79	0.10									0.62
Access to health insurance	0.38	0.07									0.14
Financial resources	0.57	0.07									0.32
Food insecurity			0.64	0.05							0.41
Household chaos			0.65	0.05							0.43
Maternal depression					0.69	0.04					0.69
Maternal anxiety					0.72	0.03					0.53
Maternal stress					0.84	0.03					0.76
Maternal experience with discrimination					0.52	0.05					0.28
Frequent family conflict							0.38	0.10			0.14
Relationship conflict							0.65	0.15			0.43
Parenting self-efficacy									0.59	0.03	0.34
Mother-child attachment									0.65	0.04	0.42
Parenting aggravation^1^									0.79	0.04	0.63
Economic Hardship^2^	--		0.27		0.09		-0.01		-0.07		
Economic Pressure			--		0.76		0.53		0.53		
Maternal Psychological Distress					--		0.62		0.74		
Relationship Problems							--		0.61		
Disrupted Parenting^3^									--		

Note. The upper-part of the table contains the standardized loadings for each indicator, and the lower-part of the table contains the correlation coefficients between the five factors. R^2^ represents the percentage of variance in each indicator that is accounted for by the latent factor

Model fit: RMSEA = 0.037, 95% CI for RMSEA = [0.020, 0.052]; CFI = 0.959, TLI = 0.943; c^2^ = 97.65, *df* = 66, *p* =.007

^1^ Unlike the other indicators included wherein higher scores represent more stress/problems, a higher score here represents lower parenting aggravation

^2^ The original Economic Hardship factor was reversed so that higher values represent *less* access to resources

^3^ The original Disrupted Parenting factor was reversed so that higher values represent *more* disrupted parenting

Upon examining the correlations among the dimensions of the model (Table 1, Supplementary Materials), several noteworthy findings emerged. First, economic hardship exhibited a mild positive correlation with economic pressure (*r* =.27), but demonstrated minimal correlation with the other dimensions of family stress. Economic pressure, on the other hand, was significantly and positively correlated with the other dimensions of family stress, with strong positive correlations with maternal psychological distress (*r* =.76), and moderate correlations with relationship problems (*r* =.53) and disrupted parenting (*r* =.53). This indicates that families experiencing higher economic pressure are also likely to face higher levels of maternal psychological distress and encounter challenges in family relationships and parenting.

Additionally, the three psychological-behavioral dimensions of family stress, namely psychological distress, relationship problems, and disrupted parenting, were highly inter-correlated. The correlations among these dimensions ranged from 0.61 to 0.74, suggesting that these aspects of family stress tend to co-occur within the families in the sample. This finding aligns with the conceptualization of the Family Stress Model, wherein these psychological and behavioral dimensions are interrelated and can collectively impact family functioning.

### Identifying Latent Profiles of Families as a Function of Family Stressors

To identify distinct patterns of family stressors across these five dimensions within our sample, we used LPA with factor scores extracted from the confirmatory factor model. The factor scores represent the five dimensions of the model: economic hardship, economic pressure, maternal psychological distress, relationship problems, and disrupted parenting. We tested models with two, three, four, and five profiles, and selected the three-profile model because the groups were distinct (detailed below) and the fit statistics were satisfactory (AIC = 4029.56, BIC = 4114.63, SSABIC = 4044.83, Entropy = 0.87).

**Fig. 1:**
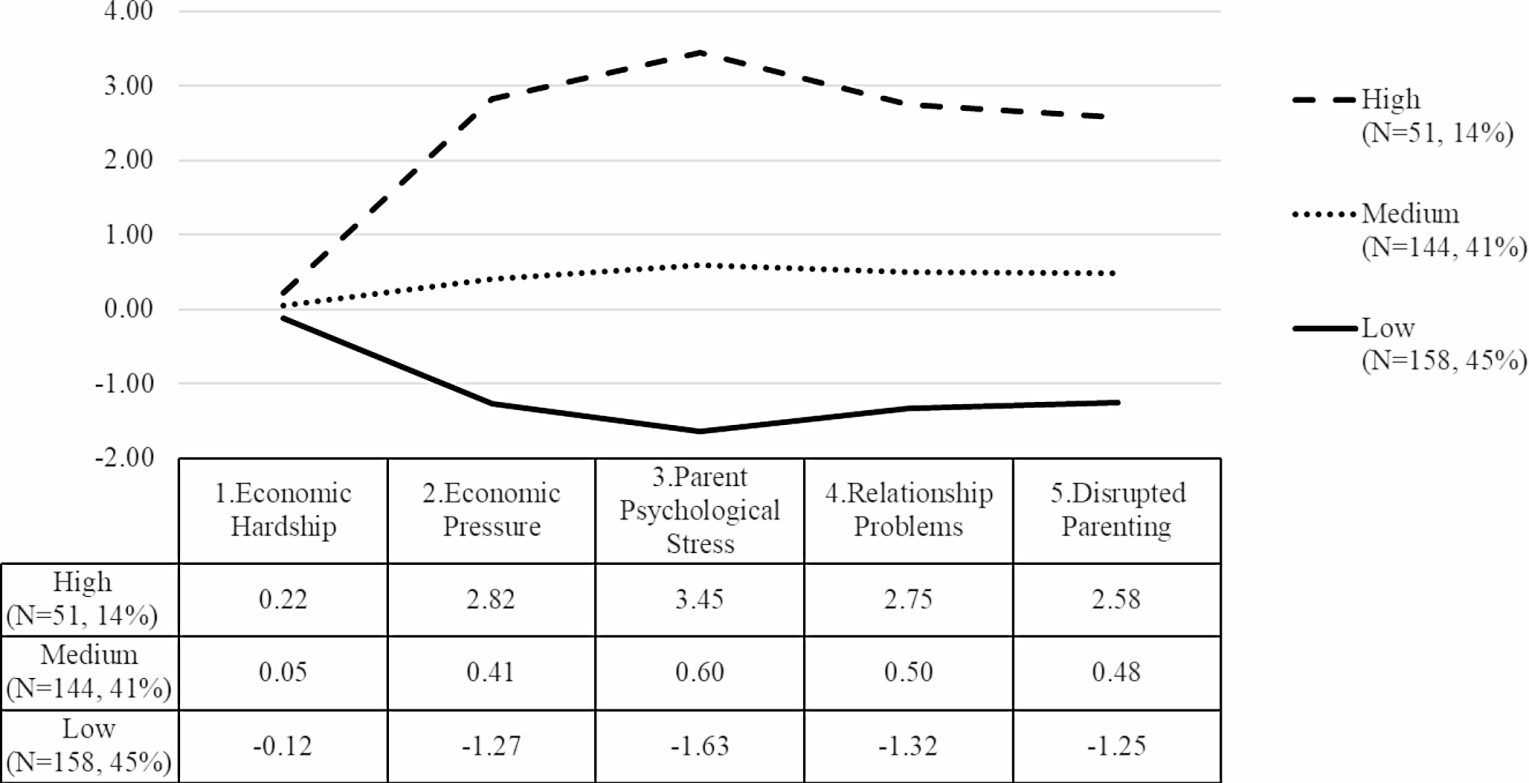
Three latent profiles of the family stress model. *Note.* The vertical axis represents the number of standard deviations from the sample mean

The three-profile solution (Fig. 1) identified the following three profiles of families: low-stress families (profile 1), medium-stress families (profile 2), and high-stress families (profile 3). Interestingly, the dimension of economic hardship, which encompassed access to resources such as income, medical insurance, and financial resources, did not exhibit significant differences among the three profiles. However, for the other four dimensions, the scores were significantly different across the profiles (*p* <.001 for all pairwise comparisons).

Among these profiles, approximately one-half (45%) of the sample belonged to the low-stress group, characterized by relatively low stress levels across the five dimensions of family stressors. Additionally, approximately 41% of the sample fell into the medium-stress category, indicating moderate stress levels encompassing various dimensions. Notably, around 14% of the sample, despite facing comparable levels of economic hardship, experienced notably higher economic pressure (2.82 *SD*s above the mean) and psychological distress (3.45 *SD*s above the mean). Moreover, these families reported higher levels of relationship problems (2.75 *SD*s above the mean) and parenting difficulties (2.58 *SD*s above the mean) compared to the other families. For full comparison of family characteristics across the three profiles, see Tables 3 and 4.

**Table 3:** Comparison of family characteristics across three latent profiles of the family stress model

						P1: Low Stress (45%)		P2: Medium Stress (41%)		P3: High Stress (14%)	*p*-value ^1^	
Child is a girl						46%		53%		51%	.509	
Child age in months (at enrollment)						7.99 (1.39)		8.02 (1.59)		8.26 (1.55)	.560	
Annual household income (in $1000)						27.10 (19.70)		24.57 (15.59)		25.40 (16.19)	.467	
Number of people in household						4.52 (1.59)		4.35 (1.45)		5.07 (1.73)	**.018**	
Primary home language is English						86%		89%		92%	.471	
Mother’s age in years						29.33 (5.48)		29.17 (5.88)		29.82 (5.84)	.781	
Mother is Hispanic						6%		8%		12%	.341	
Mother’s race											.299	
Black						51%		52%		43%		
White						36%		29%		43%		
Other (including multiracial)						13%		19%		14%		
Mother’s education											.990	
No high school diploma						14%		12%		14%		
High school diploma or GED						30%		30%		33%		
Some college or two-year degree						35%		39%		35%		
Bachelor’s degree or higher						21%		20%		18%		
Mother’s employment											.568	
Full-time						36%		37%		26%		
Part-time or self-employed						18%		20%		26%		
Not employed						46%		43%		49%		
Mother’s relationship											.056	
Married and living with a partner						39%		24%		24%		
Not married, living with a partner						20%		25%		28%		
Not living with a partner						41%		52%		48%		
Mother’s feeling of loneliness						1.74 (.45)		2.11 (.44)		2.43 (.55)	**< .001**	
Mother’s connectedness with friends						1.52 (.79)		1.50 (.69)		1.15 (.74)	**.008**	
Mother’s connectedness with organizations						.61 (.78)		.59 (.75)		.67 (.86)	.836	
Mother’s connectedness with community members						.72 (.81)		.61 (.66)		.56 (.73)	.295	
Unplanned pregnancy						55%		61%		87%	**< .001**	
Dad provides regularly						73%		71%		78%	.708	

Notes. For continuous variables, mean values were reported with standard deviation reported in parenthesis. For categorical variables, the percentage of each category was reported

^1^ We conducted ANOVA to examine differences between profiles for continuous variables, and Chi-squared tests to examine differences between profiles for categorical variables

**Table 4:** Comparison of additional child demographics and family stress indicators across three latent profiles of the family stress model

				P1: Low Stress (45%)		P2: Medium Stress (41%)		P3: High Stress (14%)	*p*-value ^1^	
Child is Hispanic				10%		9%		17%	.263	
Child’s race									.247	
Black				49%		53%		40%		
White				29%		21%		26%		
Other (including multiracial)				21%		26%		34%		
Income-to-needs ratio				1.00 (.73)		.93 (.61)		.84 (.52)	.323	
Access to health insurance									.288	
No health insurance				6%		8%		2%		
Medicaid/Government-aided insurance				73%		78%		78%		
Private or multiple insurance(s)				21%		14%		20%		
Material resources				.72 (.29)		.72 (.28)		.66 (.31)	.462	
Food insecurity				.03 (.06)		.11 (.15)		.29 (.24)	**< .001**	
Household chaos				2.15 (.50)		2.54 (.51)		3.03 (.53)	**< .001**	
Maternal depression				.17 (.22)		.64 (.57)		1.48 (.77)	**< .001**	
Maternal anxiety				.19 (.26)		.69 (.55)		1.49 (.68)	**< .001**	
Maternal stress				.88 (.50)		1.71 (.47)		2.40 (.54)	**< .001**	
Maternal experience with discrimination				.44 (.62)		.96 (.82)		1.57 (1.09)	**< .001**	
Frequent family conflict				62%		76%		90%	**< .001**	
Relationship conflict				1.90 (.37)		2.31 (.54)		2.69 (.59)	**< .001**	
Parenting self-efficacy				9.47 (.69)		8.94 (.82)		8.31 (1.13)	**< .001**	
Mother-child attachment				4.90 (.16)		4.71 (.27)		4.49 (.40)	**< .001**	
Parenting aggravation (reversed) ^2^				3.64 (.28)		3.25 (.38)		2.84 (.40)	**< .001**	

Notes. For continuous variables, mean values were reported with standard deviation reported in parenthesis. For categorical variables, the percentage of each category was reported

^1^ We conducted ANOVA to examine differences between profiles for continuous variables, and Chi-squared tests to examine differences between profiles for categorical variables

^2^ Unlike the other indicators included wherein higher scores represent more stress/problems, higher score represents lower parenting aggravation

To investigate the relationship between latent profiles of family stress and family characteristics, we explored various potential correlates that measured characteristics of the children, mothers, and families. A summary of the results is presented in Table 3. Among the 16 correlates examined, several variables exhibited significant differences among the three identified profiles, as we describe further below.

First, regarding household size, families categorized under the high-stress group reported significantly larger household sizes (median = 5) compared to families from the other two profiles. In contrast, both the low-stress and medium-stress families had median household sizes of 4 (*p* <.05). Second, mothers associated with the high-stress profile reported significantly higher levels of loneliness in comparison to mothers from the other two profiles (*p* <.001). A significant difference was also found between mothers in the low-stress and medium-stress profiles, in that the later reported higher levels of loneliness (*p* <.001). Third, mothers in the high-stress profile reported having fewer friends they can turn to for support (mean = 1.15) in contrast to mothers from the other profiles (means = 1.51 and 1.50, *p* <.05). On the other hand, the reported levels of community connection and support were not significantly different among profiles. Finally, in the high-stress profile, a substantial percentage of mothers (87%) reported that their child was an unplanned pregnancy, which was significantly higher compared to the low-stress and medium-stress profiles (55% and 61% respectively, *p* <.001).

Several additional points are worth noting. First, a number of variables, such as annual household income, maternal age, and maternal education, did not significantly differ across the profiles; this signals that such factors on their own do not reflect stressors within the household. Second, despite experiencing such factors as lower income and limited educational attainment, some households showed a low-stress profile; this may reflect resilience within the home.

## Discussion

Our study validates the Family Stress Model within a low-income sample of families with young children drawn from one midwestern state. Based on a family’s reports on14 key indicators, we identified three reliable profiles of stress within the families using this model. Notably, economic hardship is not the primary driver of profile membership within our sample, likely because all households are low-income and most of them are experiencing some degree of economic hardship; instead, variation in the other four key stressors – economic pressure, maternal psychological distress, relationship problems, and disrupted parenting – drove differences among low-, medium-, and high-stress families. Not all low-income families share the same types of stress, and certain family characteristics were connected to profile membership in the Family Stress Model. For instance, presence of larger households, higher maternal loneliness, reduced social connectedness, and increased rates of unplanned pregnancies associated with the high-stress profile warrants further investigation into how these may interact and influence family well-being. Likewise, the finding that many low-income homes were of a low-stress profile signals the importance of identifying resilience factors not examined here, such as having strong cohesion within the household (Orthner et al., 2004).

The present findings also signal the importance of determining potential linkages between family-stress profiles and children’s development. There is considerable variability among low-income children in their development, and characteristics of the household environment is associated with this variability (Chazan-Cohen et al., 2009). For example, caregiver stress is associated with children’s vocabulary skills at kindergarten entry (Chazen-Cohen et al.). Determining whether such relations reflect broader profiles of family stressors, rather than single observed variables, is an important future research direction.

A potential limitation of this study is that it uses a convenience sample, and therefore cannot be generalized to a larger population. For instance, the present sample resided in one midwestern state, and findings may not reflect family stressors commonly experienced in other geographic settings. A related limitation concerns the requirement that participating families have some comfort level in speaking English; generalizability to non English-speaking families, including recent immigrants, is unclear. A third limitation is the reliance on mother-rated measures for many of the family-stress measures. Studies that relay on other measurement approaches, such as physiological markers of stress and/or assessor-rated observations of disrupted parenting, may add more depth to our understanding of these latent variables within the Family Stress Model.

### Implications

This study provides evidence that there are low-, medium-, and high-stress profiles of family stressors within a low-income sample with young infants. The observation of reliable profiles indicates that low-income families experience varying levels of stressors and that family characteristics may, in part, determine which families experience which levels of stress. However, understanding how family characteristics and profile membership within the Family Stress Model interact and potentially influence family well-being or children’s developmental trajectories requires further study.

To further validate these findings, researchers and practitioners may examine the effects of interventions that target specific stressors on family profiles. For instance, practitioners might consider whether addressing mothers’ loneliness and connectedness might shift a family from a high-stress to a less-stressed profile over time, as these are salient to the high-stress profile. Examining stressors experienced by low-income families more holistically, as occurs at the profile-level, may present opportunities to design more personalized interventions for families experienced economic hardship.

## Electronic Supplementary Material

Below is the link to the electronic supplementary material.

Supplementary Material 1

Supplementary Material 2
