# Supplementary material for: Profiles of Family Stressors Among Low-Income Families with Young Children
Source: Matern Child Health J. 2025 Feb 25;29(4):483–93. doi: 10.1007/s10995-025-04061-2 (PMC12006244; doi:10.1007/s10995-025-04061-2)
Supplement: Supplementary file 1 — Supplementary Material 1 [file 10995_2025_4061_MOESM1_ESM.docx]

***Supplementary Materials***

***Measures and Psychometrics used to Represent Five Dimensions of the Family Stress Model and Scaled Family Characteristics***

Notably, for the originally enrolled sample (*n* =337), when data are referenced as having been collected at enrollment, this occurred at timepoint 1 of the overall study (when children were 6-12 months old). Alternatively, for the replenishment sample (*n* = 19), when data are referenced as having been collected at enrollment, this occurred at timepoint 2 of the overall study (when children were 13-15 months old), as this was the current timepoint at which the replenishment dyads joined the study.

***Economic Hardship***

Economic hardship was characterized by three measures: income-to-needs ratio, access to health insurance, and availability of financial resources. All three measures were administered at enrollment, with income-to-needs ratio and access to health insurance assessed via phone survey and availability of financial resources via emailed survey. Income-to-needs ratio was measured by first asking participants how many individuals total lived in their household. Then, we asked them to report their total annual household income for the previous year, using $10,000 increments (i.e., *$10,000 or less; $10,001-$20,000; $20,001-$30,000; …; $90,000 or more*). Finally, we calculated the income-to-needs ratio by taking the median of their reported income range and dividing that by the 2020 poverty line based on household size, such that higher scores indicate lower hardship.

Access to health insurance was measured by asking participants “what kind of health insurance or health care coverage do you have?” and allowing them to choose between the following options: *private health insurance or HMO; Medicaid; military health care/TRICARE/CHAMPUS/CHAMPVA; Indian health service; Another government program (e.g., Medicare); Other (please specify);* or *None*. We then recategorized these responses into three groups: No health insurance (0); Medicaid/Government-aided insurance (1); or Private/Multiple insurance(s) (2). Access to health insurance was treated as an ordinal variable, with higher numbers indicating more access to healthcare resources.

Availability of financial resources was measured by having participants respond *yes* or *no* to four statements about resources they (or someone in their household) may possess, including a (1) checking account, (2) savings account, (3) credit card, and (4) driver’s license (as an indicator of having access to obtain household needs). The internal consistency for this scale was acceptable within our sample (α = .65~.71). An average score was calculated for participants who answered all four items, with higher scores indicating more financial security and less hardship.

***Economic Pressure***

Economic pressure was characterized by two measures: food insecurity and household chaos. Both measures were administered via emailed survey at enrollment. Food insecurity was measured using the USDA Household Food Security Scale (Bickel et al., 2000). The USDA Food Security Scale consisted of 18 items that assess a family’s ability to meet the food needs of their family (e.g., “*In the last 12 months, were you ever hungry, but didn't eat, because there wasn't enough money for food?*”). Of these 18 items, nine were rated as *Yes* = 1 or *No* =0, while the other half were rated based on how often families faced a given problem, using either the scheme of: (a) *often* = 1*, sometimes* = 1*,* or *never* = 0 or (b) *almost every month* = 1, *some but not every month* = 1, or *only 1 or 2 months* = 0, as indicated by Bickel et al., 2000. The internal consistency for this scale was acceptable within our sample (α = 0.89~.90). An average score was calculated for participants who answered at least 15 items, with higher scores indicating lower food security, or in other words higher food insecurity.

Household chaos was measured using a shortened version of the Confusion, Hubbub, and Order Scale (CHAOS; Matheny et al., 1995). The CHAOS shortened form (first used by Petrill et al., 2004; and then by many others, as shown in Marsh, Dobson, & Maddison, 2020) consisted of six items that assess the overall feeling of the home atmosphere (e.g., “You can’t hear yourself think in our home,” and “It’s a real zoo in our home”), with participants rating each statement on a five-point scale based on how true they feel the statement is in describing their home (1 = *Definitely untrue* to 5 = *Definitely true*). The internal consistency for this scale was moderate within our sample (α = .59 at both timepoints). An average score was calculated for participants who answered at least five items, with higher scores indicating a higher level of household chaos in the home.

***Maternal Psychological Distress***

Maternal psychological distress was characterized by four measures: maternal depression, anxiety, stress, and experience with discrimination. All four measures were administered via emailed survey, with maternal depression assessed at timepoint 1, maternal anxiety at both timepoint 1 and 2, and maternal stress and experience with discrimination at timepoint 2. Maternal depression was measured using the Center for Epidemiological Studies of Depression Scale – Revised Form (CESD-R; Eaton et al., 2004; Van Dam & Earleywine, 2011). The CESD-R consisted of 20 items that assess symptoms of depression, with participants rating each statement using a five-point scale based on how often they felt that way during the last two week (0 = *Not at all or less than 1 day* to 4 = *Nearly every day*). The internal consistency for this scale was acceptable within our sample (α = .94). An average score was calculated for participants who answered at least 16 items, with higher scores indicating higher prevalence of depressive symptoms.

Maternal anxiety was measured using the Generalized Anxiety Disorder 7-Item Scale (GAD-7; Löwe et al., 2008; Spitzer et al., 2006). The GAD-7 consisted of seven items that assess symptoms of anxiety, with participants rating each statement using a four-point scale based on how often they felt that way during the last two weeks (0 = *Not at all* to 3 = *Nearly every day*). The internal consistency for this scale was acceptable within our sample (α = .92~.93). An average score was calculated for participants who answered at least 6 items, with higher scores indicating higher prevalence of anxiety symptoms.

Maternal stress was measured using the Perceived Stress Scale (PSS; Cohen, 1994; Cohen et al., 1983). The PSS consisted of 10 items that assess the participant’s perception of stress experienced in their day-to-day life, with participants rating each statement using five-point scale based on how often they felt that way during the last month (0 = *Never* to 4 = *Very often*). The internal consistency for this scale was acceptable within our sample (α = .87). An average score was calculated for participants who answered at least 8 items, with higher scores indicating higher prevalence of perceived stress.

Maternal experience with discrimination was measured using the Everyday Discrimination Scale (EDS; Williams et al., 1997). The EDS consisted of nine items that assess how often the participant experiences different topics of discrimination, with participants rating each statement on a six-point scale based on how often they experienced something negative related to each topic (0 = *Never* to 5 = *Almost every day*). The internal consistency for this scale was acceptable within our sample (α = .89). An average score was calculated for participants who answered at least eight items, with higher scores indicating higher perceived experience with discrimination.

***Relationship Problems***

Relationship problems was characterized by two measures: family conflict and relationship conflict. Both measures were administered at enrollment via emailed survey. Family conflict was measured using the Family Environment Scale – Conflict Subscale (FES; Moos, 1974; Moos & Moos, 2009). The FES Conflict Subscale consisted of nine items that assess family conflict and negative communication, with participants rating each statement on *true* or *false* based on whether the statement frequently describes their family. The internal consistency for this scale was acceptable within our sample (α = .70~.77). A sum score was calculated for participants who answered at least eight items, with higher scores indicating higher family conflict. Prior to analyses, the scores for this scale were dichotomized, such that 1 = frequent family conflict indicated as “true” for at least one of the eight items and 0 = false to all items, as the original sum score was highly skewed.

Relationship conflict was measured using a subset of questions from the Braiker-Kelley Relational Intimacy Scale – Conflict-Negativity Subscale (Braiker & Kelley, 1979). The modified Conflict-Negativity Subscale consisted of four items (the first four questions from the original five-item scale) that assess behavioral conflict and negative communication, with participants rating each statement on a five-point scale based on how often they exhibit these behaviors within their relationship (1 = *Never* to 5 = *Always*). The internal consistency for this scale was acceptable within our sample (α = .65~.66). An average score was calculated for participants who answered all 4 items, with higher scores indicating higher relationship conflict.

***Disrupted Parenting***

Disrupted parenting was characterized by three measures: parenting self-efficacy, mother-child attachment, and parenting aggravation. All three measures were administered at enrollment via emailed survey. Parenting self-efficacy was measured using the Parental Cognitions and Conduct Toward the Infant Scale – Parental Self-Efficacy Subscale (PACOTIS; Boivin et al., 2005). The Parental Self-Efficacy Subscale consisted of six items that assess the perception of the mother’s feelings of effectiveness as a parent, with participants rating each statement on an 11-point scale based on how well each statement describes them (0 = *Not at all true* to 10 = *Completely true*). The internal consistency for this scale was acceptable within our sample (α = .78~.87). An average score was calculated for participants who answered at least five items, with higher scores indicating higher perceived effectiveness.

Mother-child attachment was measured using the Maternal Postnatal Attachment Questionnaire – Quality of Attachment Subscale (MPAQ; Condon & Corkindale, 1998). The MPAQ Quality of Attachment Subscale consisted of nine items that assess the quality of attachment between a mother and her child, with participants rating each statement based on how they felt the statement fit their relationship with their child (responses/scores vary by question, but follow the guidelines set by Condon & Corkindale, 1998; scores range from 1 to 5). The internal consistency for this scale was moderate within our sample (α = .53~.73). An average score was calculated for participants who answered at least eight items, with higher scores indicating a higher quality of mother-child attachment.

Parenting aggravation was measured using the Fragile Families Aggravation in Parenting Questions (The Fragile Families and Child Wellbeing Survey, 2005). The Aggravation in Parenting Questions consisted of nine items that assess how parenting makes the participant feel, with participants rating each statement on a four-point scale based on how much they agree (1 = *Strongly agree* to 4 = *Strongly disagree*) with each statement. The internal consistency for this scale was acceptable within our sample (α = .72~.75). An average score was calculated for participants who answered at least eight items, with lower scores indicating higher aggravation in parenting and higher scores indicating lower aggravation in parenting.

***Family Characteristics with Scale Scores***

Mother’s social connectedness with friends and mother’s social connectedness with community were measured at enrollment via emailed survey using questions adapted from the Berkman-Syme Social Network Index (SNI; Berkman, 1977; Berkman & Syme, 1979). To measure mother’s social connectedness with friends, participants rated three questions adapted from the SNI, which asked about (1) the number of adult friends in their life, (2) the number of adult friends who can be counted on for providing physical or financial support (e.g., providing a loan, childcare, or transportation support), and (3) the number of adult friends who can be counted on for providing emotional support, using a five-point scale (0 = *None* to 4 = *More than 10*). The internal consistency for this scale was high within our sample (α = .86~.90). An average score was calculated for participants who answered any of the three questions, with higher scores indicating higher connectedness with friends.

To measure mother’s social connectedness with community, participants rated two questions adapted from the SNI, which asked about (1) the number of social or community organizations participated in regularly once a week or more and (2) the number of social or community organizations participated in occasionally or infrequently, using a six-point scale (0 = *None* to 5 = *More than 10*). Reliability for the subscale of “connectedness with organizations” was high within our sample (α = .84~.86). Additionally, participants rated two more questions adapted from the SNI, which asked about (1) the number of community members who can be counted on for providing physical or financial support (e.g., providing a loan, childcare, or transportation support) and (2) the number of community members who can be counted on for providing emotional support, using a five-point scale (0 = *None* to 4 = *More than 10*). The internal consistency for this subscale of “connectedness with community members” was high in our sample (α = .84~.87). For each subscale, an average score was calculated for participants who answered at least one question, with higher scores indicating higher connectedness with community.

Finally, mother’s loneliness was measured at timepoint 2 via emailed survey using the UCLA Loneliness Scale version 3 (Russell, 1996). This scale consisted of 20 items that assess subjective feelings of social isolation and loneliness, with participants rating each statement using a four-point scale based on how much they agree (1 = *Strongly disagree* to 4 = *Strongly agree*) with each statement. The internal consistency for this scale was acceptable within our sample (α = .95). An average score was calculated for participants who answered at least 16 items, with higher scores indicating higher perceived loneliness.

**References**

Berkman, L. F. (1977). *Social networks, host resistance, and mortality: A follow-up study of Alameda County residents* [Doctoral dissertation, University of California, Berkeley].

Berkman, L. F., & Syme, S. L. (1979). Social networks, host resistance, and mortality: a nine-year follow-up study of Alameda County residents. *American Journal of Epidemiology*, *109*(2), 186-204. https://doi.org/10.1093/oxfordjournals.aje.a112674

Bickel, G., Nord, M., Price, C., Hamilton, W., & Cook, J. (2000). *Measuring Food Security in the United States: Guide to measuring household food security, revised 2000*. https://ageconsearch.umn.edu/record/337157/

Boivin, M., Pérusse, D., Dionne, G., Saysset, V., Zoccolillo, M., Tarabulsy, G. M., Tremblay, N., & Tremblay, R. E. (2005). The genetic‐environmental etiology of parents' perceptions and self‐assessed behaviours toward their 5‐month‐old infants in a large twin and singleton sample. *Journal of Child Psychology and Psychiatry*, *46*(6), 612-630. https://doi.org/10.1111/j.1469-7610.2004.00375.x

Braiker, H., & Kelley, H. (1979). Conflict in the development of close relationships. In R. Burgess & T. Huston (Eds.), *Social exchange in developing relationships* (pp. 135-168). Academic Press.

Cohen, S. (1994). *Perceived stress scale - Measuring stress: A guide for health and social scientists*. https://www.northottawawellnessfoundation.org/wp-content/uploads/2018/04/PerceivedStressScale.pdf

Cohen, S., Kamarck, T., & Mermelstein, R. (1983). A global measure of perceived stress. *Journal of Health and Social Behavior*, *24*(4), 386-396. https://doi.org/10.2307/2136404

Condon, J. T., & Corkindale, C. J. (1998). The assessment of parent-to-infant attachment:Development of a self-report questionnaire instrument. *Journal of Reproductive and Infant Psychology*, *16*(1), 57-76. https://doi.org/10.1080/02646839808404558

Eaton, W. W., Smith, C., Ybarra, M., Muntaner, C., & Tien, A. (2004). Center for Epidemiologic Studies Depression Scale: review and revision (CESD and CESD-R). In M. E. Maruish (Ed.), *The Use of Psychological Testing for Treatment Planning and Outcomes* (3rd ed., pp. 363-377). Lawrence Erlbaum.

The Fragile Families and Child Wellbeing Survey. (2005). *Fragile Families: Scales documentation and question sources for one-year questionnaires*. http://www.fragilefamilies.princeton.edu/surveys/Fragile%20Families%20One-Year%20Scales%20Documentation%20-jk%20092905.pdf

Löwe, B., Decker, O., Müller, S., Brähler, E., Schellberg, D., Herzog, W., & Herzberg, P. Y. (2008). Validation and standardization of the Generalized Anxiety Disorder Screener (GAD-7) in the general population. *Medical Care*, 266-274. https://www.jstor.org/stable/40221654

Marsh, S., Dobson, R., & Maddison, R. (2020). The relationship between household chaos and child, parent, and family outcomes: A systemic scoping review. *BMC Public Health*, *20*, 513. https://doi.org/10.1186/s12889-020-08587-8

Matheny, A. P., Wachs, T. D., Ludwig, J. L., & Phillips, K. (1995). Bringing order out of chaos: Psychometric characteristics of the confusion, hubbub, and order scale. *Journal of Applied Developmental Psychology*, *16*(3), 429-444. https://doi.org/10.1016/0193-3973(95)90028-4

Moos, R. H. (1974). *Family environment scale preliminary manual*. Consulting Psychologists Press.

Moos, R. H., & Moos, B. S. (2009). *Family environment scale manual: Development, applications and research* (4th ed.). Mind Garden Inc.

Petrill, S. A., Pike, A., Price, T., & Plomin, R. (2004). Chaos in the home and socioeconomic status are associated with cognitive development in early childhood: Environmental mediators identified in a genetic design. *Intelligence*, *32*(5), 445-460. https://doi.org/10.1016/j.intell.2004.06.010

Russell, D. W. (1996). UCLA Loneliness Scale (version 3): Reliability, validity, and factor structure. *Journal of Personality Assessment*, *66*(1), 20-40. https://doi.org/10.1207/s15327752jpa6601_2

Spitzer, R. L., Kroenke, K., Williams, J. B., & Löwe, B. (2006). A brief measure for assessing generalized anxiety disorder: the GAD-7. *Archives of Internal Medicine*, *166*(10), 1092-1097. https://doi.org/10.1001/archinte.166.10.1092

Van Dam, N. T., & Earleywine, M. (2011). Validation of the Center for Epidemiologic Studies Depression Scale—Revised (CESD-R): Pragmatic depression assessment in the general population. *Psychiatry Research*, *186*(1), 128-132. https://doi.org/10.1016/j.psychres.2010.08.018

Williams, D. R., Yu, Y., Jackson, J. S., & Anderson, N. B. (1997). Racial differences in physical and mental health: Socioeconomic status, stress, and discrimination. *Journal of Health Psychology*, *2*(3), 335-351. https://doi.org/10.1177/135910539700200305
