# Supplementary material for: Profiles of Family Stressors Among Low-Income Families with Young Children
Source: Matern Child Health J. 2025 Feb 25;29(4):483–93. doi: 10.1007/s10995-025-04061-2 (PMC12006244; doi:10.1007/s10995-025-04061-2)
Supplement: Supplementary file 2 — Supplementary Material 2 [file 10995_2025_4061_MOESM2_ESM.docx]

Supplemental Table 1

*Correlation coefficients among indicators of the Family Stress Model*

|  | 1 | 2 | 3 | 4 | 5 | 6 | 7 | 8 | 9 | 10 | 11 | 12 | 13 | 14 |
| --- | --- | --- | --- | --- | --- | --- | --- | --- | --- | --- | --- | --- | --- | --- |
| 1. Income-to-needs ratio | -- | 0.26 | 0.44 | -0.13 | -0.12 | -0.07 | -0.11 | -0.09 | -0.02 | -0.09 | 0.06 | -0.05 | -0.04 | -0.01 |
| 2. Access to health insurance |  | -- | 0.22 | -0.06 | 0.00 | -0.02 | -0.05 | -0.02 | 0.04 | -0.05 | -0.02 | -0.06 | -0.03 | -0.03 |
| 3. Financial resources |  |  | -- | -0.17 | -0.07 | 0.01 | -0.00 | -0.06 | 0.01 | -0.04 | 0.09 | -0.01 | -0.06 | -0.03 |
| 4. Food insecurity |  |  |  | -- | 0.41 | 0.30 | 0.32 | 0.41 | 0.36 | 0.08 | 0.20 | -0.22 | -0.13 | -0.30 |
| 5. Household chaos |  |  |  |  | -- | 0.31 | 0.31 | 0.46 | 0.24 | 0.17 | 0.22 | -0.26 | -0.15 | -0.36 |
| 6. Maternal depression |  |  |  |  |  | -- | 0.75 | 0.52 | 0.32 | 0.10 | 0.31 | -0.29 | -0.41 | -0.42 |
| 7. Maternal anxiety |  |  |  |  |  |  | -- | 0.64 | 0.39 | 0.09 | 0.30 | -0.28 | -0.37 | -0.42 |
| 8. Maternal stress |  |  |  |  |  |  |  | -- | 0.46 | 0.15 | 0.34 | -0.37 | -0.32 | -0.49 |
| 9. Maternal experience with discrimination |  |  |  |  |  |  |  |  | -- | 0.15 | 0.14 | -0.15 | -0.18 | -0.33 |
| 10. Frequent family conflict |  |  |  |  |  |  |  |  |  | -- | 0.18 | -0.05 | -0.04 | -0.16 |
| 11. Relationship conflict |  |  |  |  |  |  |  |  |  |  | -- | -0.16 | -0.35 | -0.36 |
| 12. Parenting self-efficacy |  |  |  |  |  |  |  |  |  |  |  | -- | 0.48 | 0.41 |
| 13. Mother-child attachment |  |  |  |  |  |  |  |  |  |  |  |  | -- | 0.44 |
| 14. Parenting aggravation^1^ |  |  |  |  |  |  |  |  |  |  |  |  |  | -- |

*Note*. Pearson correlation coefficients were calculated for correlations between two continuous variables. Spearman correlation coefficients were calculated for correlations between two ordinal variables, or between an ordinal variable and continuous variables.

^1^ Unlike the other indicators included wherein higher scores represent more stress/problems, a higher score here represents lower parenting aggravation.
